# Treatment Failure and Mortality amongst Children with Severe Acute Malnutrition Presenting with Cough or Respiratory Difficulty and Radiological Pneumonia

**DOI:** 10.1371/journal.pone.0140327

**Published:** 2015-10-09

**Authors:** Mohammod Jobayer Chisti, Mohammed Abdus Salam, Pradip Kumar Bardhan, Abu S. G. Faruque, Abu S. M. S. B. Shahid, K. M. Shahunja, Sumon Kumar Das, Md Iqbal Hossain, Tahmeed Ahmed

**Affiliations:** 1 Centre for Nutrition & Food Security (CNFS), International Centre for Diarhoeal Disease Research, Bangladesh (icddr,b), Dhaka, Bangladesh; 2 Dhaka Hospital, icddr,b, Dhaka, Bangladesh; 3 Research & Clinical Administration and Strategy (RCAS), icddr,b, Dhaka, Bangladesh; Instituto de Higiene e Medicina Tropical, PORTUGAL

## Abstract

**Background:**

Appropriate intervention is critical in reducing deaths among under-five, severe acutely malnourished (SAM) children with danger signs of severe pneumonia; however, there is paucity of data on outcome of World Health Organisation (WHO) recommended interventions of SAM children with severe pneumonia. We sought to evaluate outcome of the interventions in such children.

**Methods:**

We prospectively enrolled SAM children aged 0–59 months, admitted to the Intensive Care Unit (ICU) or Acute Respiratory Infection (ARI) ward of the Dhaka Hospital of the International Centre for Diarrhoeal Disease Research, Bangladesh (icddr,b), between April 2011 and June 2012 with cough or respiratory difficulty and radiological pneumonia. All the enrolled children were treated with ampicillin and gentamicin, and micronutrients as recommended by the WHO. Comparison was made among pneumonic children with (n = 111) and without WHO defined danger signs of severe pneumonia (n = 296). The outcomes of interest were treatment failure (if a child required changing of antibiotics) and deaths during hospitalization. Further comparison was also made among those who developed treatment failure and who did not and among the survivors and deaths.

**Results:**

SAM children with danger signs of severe pneumonia more often experienced treatment failure (58% vs. 20%; p<0.001) and fatal outcome (21% vs. 4%; p<0.001) compared to those without danger signs. Only 6/111 (5.4%) SAM children with danger signs of severe pneumonia and 12/296 (4.0%) without danger signs had bacterial isolates from blood. In log-linear binomial regression analysis, after adjusting for potential confounders, danger signs of severe pneumonia, dehydration, hypocalcaemia, and bacteraemia were independently associated both with treatment failure and deaths in SAM children presenting with cough or respiratory difficulty and radiological pneumonia (p<0.01).

**Conclusion and Significance:**

The result suggests that SAM children with cough or respiratory difficulty and radiologic pneumonia who had WHO-defined danger signs of severe pneumonia more often had treatment failure and fatal outcome compared to those without the danger signs. In addition to danger signs of severe pneumonia, other common causes of both treatment failure and deaths were dehydration, hypocalcaemia, and bacteraemia on admission. The result underscores the importance for further research especially a randomized, controlled clinical trial to validate standard WHO therapy in SAM children with pneumonia especially with danger signs of severe pneumonia to reduce treatment failures and deaths.

## Introduction

Many developing countries have reported 25% reductions in pneumonia episodes per child-year over the past decade [1], yet pneumonia remains the leading cause of childhood mortality globally [2, 3]. A recent systematic review has suggested that most of the pneumonic deaths were attributable to severe episodes of pneumonia in children under five who were hospitalized during 2010–2011 [1]. The deaths were disproportionately higher among severe acutely malnourished (SAM) children, particularly in sub-Saharan Africa and in South-East Asia [4–6]. A recent Lancet Nutrition Series reported that 15% of global and 16.7% of the South-East Asian childhood pneumonia deaths were attributable to severe wasting [5, 7, 8]. Sixty-seven percent (67%) of the deaths due to severe pneumonia were reported to occur early during the course of illness, i.e. in the first 48–72 hours [9]. The appropriate management during this period, including early administration of appropriate antibiotics, is critical and has been shown to dramatic reduction of deaths [10]. Ideally, the selection of antibiotic(s) should be based primarily on the underlying bacterial aetiology of severe pneumonia among the target population. Prior to the introduction of conjugate vaccine in many lower and middle income countries (LMICs) *Streptococcus pneumoniae* and *Haemophilus influenzae* type B were the most important primary bacterial pathogens and were associated with 33% and 16% of the pneumonic deaths respectively [1]. Consequently, the World Health Organisation (WHO) recommends the use of parenteral penicillin or ampicillin and gentamicin in addition to micronutrients for treatment of severe pneumonia in young children [4, 11, 12]. However, after introduction of pneumococcal and *Haemophilus influenzae* type b vaccines in LMICs with greatest burden of childhood pneumonia, bacterial aetiology of pneumonia is yet not available. Pneumonia Etiology Research for Child Health (PERCH) project, accomplished in 7 LMICs, aims to provide comprehensive data on overall aetiology that reflect the epidemiologic situation in developing countries in 2015 [13]. Till date, studies evaluating the aetiology of pneumonia in young children have largely been conducted in generalized populations and have not focused specifically on children with SAM [1, 13]. Recent data suggest that the range of bacterial pathogens causing pneumonia in SAM children is different; Gram negative bacteria play a much more significant role and are also associated with higher deaths [4, 14]. Additionally, organisms, especially gram-negatives causing severe pneumonia in these children are often resistant to penicillin and ampicillin, and also gentamicin, but have better susceptibility to extended spectrum cephalosporin such as ceftriaxone and also to fluoroquinolones, including ciprofloxacin [15, 16]. Penetration of ceftriaxone and ciprofloxacin in the pneumonic lung is also considerably better than that of ampicillin or gentamicin [17]. WHO guidelines do not differentiate antibiotic treatment for hospitalised SAM children (penicillin/ampicillin and gentamicin) with those who have pneumonia, and also with those who have danger signs of severe pneumonia, such as hypoxemia, cyanosis, grunting, convulsion, inability to drink or persistent vomiting [18]. The WHO guidelines recommend that children with severe pneumonia who fail to respond to these initially administered antibiotics may be treated with ceftriaxone [18]. The switching-over to ceftriaxone only after treatment failure in SAM children with danger signs of severe pneumonia may result in significant delay in appropriate treatment and leading to poor outcomes.

To our knowledge, there is no data on the outcomes (treatment failure and deaths) and potential risk factors of outcomes of WHO recommended interventions of hospitalised pneumonic children with SAM presenting with danger signs of severe pneumonia compared to those without danger signs of severe pneumonia. We conducted this prospective, observational study to address these issues in SAM children with severe pneumonia.

## Materials and Methods

### Ethics statement

This research study was approved by the institutional review boards of the International Centre for Diarrhoeal Disease Research, Bangladesh (icddr,b), namely the Research Review Committee (RRC) and the Ethical Review Committee (ERC). A written informed consent was obtained from attending parents or caregivers of each of the participating children; children were not enrolled if their attending parents or caregivers did not provide the consent.

### Study Setting

The study was conducted at the Dhaka Hospital of icddr,b; description of this hospital has been provided elsewhere [19].

### Study design

This was a prospectively conducted observational study. In this study, we enrolled SAM children of either sex, aged 0–59 months, with cough or respiratory difficulty and had X-ray proven pneumonia, who were admitted into the Intensive Care Unit (ICU) or Acute Respiratory Infection (ARI) ward of the Dhaka Hospital of icddr,b between April 2011 and June 2012. All the study children had both WHO defined clinical as well as radiological pneumonia. On admission, all the study children (with nor without danger signs of severe pneumonia) had received parenteral ampicillin and gentamicin, and micronutrients in adequate dose and duration following WHO guideline [18]. Comparison of socio-demographic, clinical, and laboratory characteristics on admissions and outcomes of the WHO recommended interventions in pneumonic children having WHO defined danger signs of severe pneumonia was made with those without danger signs. Further comparison of admission characteristics was also made among study children who developed treatment failure and who did not and among the survivors and deaths.

The WHO defined danger signs of severe pneumonia include hypoxemia, cyanosis, grunting respiration, convulsion, inability to drink or persistent vomiting [18]. SAM was defined in a recently published article mainly following WHO anthropometry guidelines [19], and radiological pneumonia according to WHO recommended radiological classification of pneumonia [20].

Our outcomes of interest were treatment failure and deaths of the study children during their hospitalisation. Treatment failure was considered if a child required changing of the initially administered antibiotics per WHO guideline of management of pneumonia [18]. The initial antibiotic therapy was changed if there was: (i) significant clinical deterioration, as defined by the development of any new danger signs or signs of sepsis/severe sepsis within 24 hours of receiving ampicillin and gentamicin, or (ii) persistence of danger signs of severe pneumonia even after 48 hours of initiation of the therapy. All the study children who died had initial consequence of treatment failure during hospitalization. We defined sepsis and severe sepsis in our recently published article [21].

### Patient management

All children (with or without danger signs of severe pneumonia) received WHO recommended care and treatment for hospitalized SAM children with pneumonia including parenteral ampicillin and gentamicin, micronutrients, nasogastric or oral feeding [18] or appropriate intravenous fluids if the child had severe respiratory distress; and hourly monitoring of clinical signs of respiratory distress and of SpO_2_. The study children also received treatment for co-morbidities, if any, including sepsis/severe sepsis according to the hospital guideline [21]. For children assessed to have treatment failure, antibiotics were changed to the second line agents (combination of ceftriaxone and levofloxacin) following the hospital protocol [22]. Other supportive care received by the children in this study have been described elsewhere [19].

### Measurements

Case Report Forms (CRF) were developed, pretested, and finalized for acquisition of study relevant data. Data collected on the children included their socio-demographic information (age, sex, socio-economic status, residence, mothers employment status, history of breastfeeding since neonatal period); immunization status; anthropometric information such as weight for age Z score, weight for length/height Z score, mid upper arm circumference; clinical characteristics such as duration and type of diarrhoea, dehydration status, presence of fever and its duration, laboratory test results such as hypoglycaemia (RBS <3.0 mMol/L), severe anaemia (haematocrit <15%), bacteraemia (isolation of bacterial pathogen from blood sample culture performed only once), hypokalaemia (serum potassium < 3.5 mMol/L), hyperkalaemia (serum potassium > 5.5 mMol/L), hyponatraemia (serum sodium < 130.0 mMol/L), hypocalcaemia (serum calcium < 2.12 mMol/L), hypomagnesaemia (serum magnesium < 0.7 mMol/L) on admission; severe sepsis and outcomes such as treatment failure and deaths during hospitalisation. All these information, with the exception of treatment failure and deaths, represent admission characteristics of the enrolled children.

### Analysis

All data were entered into SPSS for Windows (version 17.0; SPSS Inc, Chicago) and Epi-Info (version 6.0, USD, Stone Mountain, GA). Differences in proportion were compared by the Chi-square test. Student’s t-test was used to compare the means of normally distributed data and Mann-Whitney test was used for comparison of data that were not normally distributed. A probability of less than 0.05 was considered statistically significant. Strength of association was determined by calculating odds ratio (OR) or relative risks (RR) and their 95% confidence intervals (CIs). In identifying independent associated factors with treatment failure and deaths, variables were initially analyzed in a uni-variate model, and then risk factors independently associated separately with treatment failure and deaths were identified using log-linear binomial regression controlling for the co-variates. We have also performed sensitivity, specificity, PPV, and NPV of characteristics those were significantly associated with treatment failure and deaths by the log-linear binomial regression analyses.

## Results

In total, 1482 SAM children under five were admitted to the Dhaka Hospital of icddr,b during the study period. Out of them, 407 fulfilled our inclusion criteria of which 111 (27%) had danger signs of severe pneumonia and 296 (63%) did not have danger signs. Study children with danger signs of severe pneumonia more often presented with fever, dehydration, severe sepsis, hypokalaemia, hypocalcaemia, and hypomagnesaemia on admission compared to those without danger signs (Table 1). Only 2 of our study children having danger signs and 4 without danger signs of severe pneumonia had a blood culture isolate that were not susceptible to ampicillin and gentamicin. On the other hand, 3 study children with a blood culture isolate were not susceptible to ceftraixone and only one children to ciprofloxacin. However, overall 18 (4.4%) children had bacteraemia and the difference of bacteraemia among the groups was not significant (Table 1). A total of 67 (16.5%) children had history of prior use of antibiotics and among them only 2 (3%) children had bacteraemia. Other characteristics shown in Table 1 were also comparable between the groups.

**Table 1:** Socio-demographic, clinical and laboratory characteristics of under-five, severely malnourished children with danger signs of severe pneumonia on admission who received standard WHO interventions and those without danger signs of severe pneumonia.

Characteristic	Study children with danger signs of severe pneumonia (n = 111)	Study children without danger signs of severe pneumonia (n = 296)	OR	95% CI	p
Male sex	55 (50)	175 (59)	0.68	0.43–1.08	0.105
Age in months (median, IQR)	7.3 (4.0, 18.0)	10.0 (5.1, 18.0)	-	-	0.197
Live outside Dhaka district	21 (19)	70 (24)	0.75	0.42–1.34	0.375
Live in slum area	55 (50)	131 (44)	1.24	0.78–1.96	0.399
Poor socio-economic condition	93 (84)	251 (85)	0.93	0.49–1.76	0.922
Employed mother	15 (13)	46 (15)	0.85	0.43–1.66	0.726
Lack of intake of BCG vaccination	18 (16)	33 (11)	1.54	0.79–2.99	0.227
Lack of intake of DPT/oral polio/HIV/Hepatitis vaccination	29 (26)	53 (18)	1.62	0.94–2.80	0.089
Non-breastfed from neonatal period	22 (20)	41 (14)	1.54	0.83–2.82	0.184
Weight for length Z score	-4.0 ± 1.7	-3.8 ± 1.3	-0.29*	-0.65–0.07	0.119
Weight for age Z score	-5.1 ± 1.6	-5.0 ± 1.5	-0.11*	-0.45–0.22	0.509
Mid upper arm circumference	10.4 ± 1.6	10.5 ± 1.2	-0.12*	-0.49–0.24	0.512
AWD	89 (80)	217 (73)	1.47	0.84–2.60	0.194
Fever	76 (69)	156 (53)	1.95	1.20–3.17	0.006
Dehydration	27 (24)	25 (8)	3.48	1.84–6.60	<0.001
Duration of fever	4.0 (3.0, 5.0)	5.0 (3.0, 7.0)	-	-	0.150
Severe sepsis	11 (10)	9 (3)	6.19	2.81–13.81	0.008
Haematocrit (%)	30.7 ± 7.6	31.4 ± 5.6	-0.73*	-2.29–0.84	0.360
Overall bacteraemia	6 (5)	12 (4)	1.34	0.44–3.98	0.592
Hypokalaemia	48 (43)	91 (31)	1.72	1.07–2.76	0.024
Hyperkalaemia	14 (13)	40 (14)	.87	0.31–2.27	0.935
Hyponatraemia	18 (16)	44 (15)	1.11	0.58–2.09	0.855
Hypocalcaemia	41 (37)	59 (20)	2.35	1.42–3.91	<0.001
Hypomagnesaemia	7 (6)	5 (2)	3.92	1.09–14.57	0.021

Figures represent n (%), unless specified. OR: odds ratio. CI: confidence interval. IQR: inter-quartile range

*: mean difference

Study children with danger signs of severe pneumonia more often developed treatment failure and had fatal outcome compared to those without danger signs (Table 2). On the other hand, study children with treatment failure more often had fatal outcome compared to those without treatment failure [25 (20%) vs. 10 (4%); OR = 6.99; 95% CI = 3.07–16.23; p<0.001].

**Table 2:** Outcome of the World Health Organization recommended interventions in hospitalised, severely malnourished, under-five children with danger signs of severe pneumonia and those without danger signs of severe pneumonia.

Characteristic	Study children with danger signs of severe pneumonia (n = 111)	Study children without danger signs of severe pneumonia (n = 296)	RR	95% CI	p
Treatment failure	64 (58)	59 (20)	3.14	2.30–4.29	<0.001
Deaths	23 (21)	12 (4)	2.78	2.06–3.75	<0.001

RR: relative risks.

On the basis of the results shown in Table 3 log-linier binomial regression analyses (Table 4) was done to evaluate independent risk factors for treatment failure and those include admission danger signs of severe pneumonia, fever, dehydration, severe sepsis, hypocalcaemia and bacteraemia. On the basis of the results shown in Table 5 in a similar analysis in Table 6, the independent risk factors for deaths were almost same except fever and severe sepsis, however, employed mother was identified as an additional independent risk factor for deaths.

**Table 3:** Factors present on admission that were associated with treatment failure during hospitalisation in under-five, severely malnourished children presenting with cough or respiratory difficulty and radiological pneumonia.

Characteristic	Treatment failure (n = 123)	Without treatment failure (n = 284)	RR	95% CI	p
Male sex	67 (55)	163 (57)	0.92	0.68–1.24	0.662
Age in months (median, IQR)	8.0 (5.0, 16.0)	10.0 (5.0, 18.0)	-	-	0.772
Live outside Dhaka district	29 (24)	62 (22)	1.07	0.76–1.51	0.796
Live in slum area	59 (48)	127 (45)	1.10	0.82–1.47	0.620
Poor socio-economic condition	105 (85)	239 (84)	1.07	0.70–1.63	0.872
Employed mother	18 (15)	43 (15)	0.97	0.64–1.48	0.984
Lack of intake of BCG vaccination	13 (11)	38 (13)	0.82	0.50–1.35	0.533
Lack of intake of DPT/oral polio/HIV/Hepatitis vaccination	31 (25)	51 (18)	1.34	0.96–1.85	0.124
Non-breastfed from neonatal period	21 (17)	42 (15)	1.12	0.76–1.65	0.663
Weight for length Z score	-3.9 ± 1.4	-3.8 ± 1.5	-0.11*	-0.42–0.20	0.496
Weight for age Z score	-4.8 ± 1.9	-5.1 ± 1.3	-0.23*	-0.16–0.61	0.247
Mid upper arm circumference	10.6 ± 1.4	10.5 ± 1.3	-0.09*	-0.22–0.41	0.566
AWD	88 (72)	218 (77)	0.84	0.61–1.15	0.320
Fever	88 (72)	144 (51)	1.90	1.35–2.66	<0.001
Dehydration	27 (22)	25 (9)	1.92	1.40–2.62	<0.001
Duration of fever	4.0 (3.0, 6.0)	4.0 (3.0, 7.0)	-	-	0.900
Presence of danger signs	64 (52)	47 (17)	2.89	2.19–3.82	<0.001
Severe sepsis	11 (9)	9 (3)	1.92	1.25–2.94	0.023
Severe anaemia	4 (11)	0	3.39	2.91–3.94	0.008
Overall bacteraemia	10 (8)	8 (3)	1.90	1.22–2.96	0.034
Hypokalaemia	46 (37)	92 (32)	1.15	0.85–1.56	0.427
Hyperkalaemia	21 (17)	33 (12)	1.35	0.93–1.95	0.183
Hyponatraemia	22 (18)	40 (14)	1.21	0.83–1.76	0.407
Hypocalcaemia	46 (37)	54 (19)	1.83	1.38–2.44	<0.001
Hypomagnesaemia	7 (6)	5 (2)	1.95	1.18–3.22	0.052

Figures represent n (%), unless specified. RR: relative risks. CI: confidence interval. IQR: inter-quartile range

*: mean difference.

**Table 4:** Results of log-linear binomial regression, to identify independent risk factors for treatment failure in SAM children presenting with cough or respiratory difficulty and radiological pneumonia and who received standard WHO interventions.

Characteristic	RR	95% CI	p value
Documented fever	1.63	1.22–2.18	0.001
Dehydration	1.59	1.25–2.02	<0.001
Presence of danger signs	2.34	1.76–3.12	<0.001
Severe sepsis	1.47	1.19–1.81	<0.001
Hypocalcaemia	1.47	1.13–1.92	0.004
Bacteraemia	1.88	1.51–2.33	<0.001

**Table 5:** Factors present on admission that were associated with deaths during hospitalisation in under-five, severely malnourished children presenting with cough or respiratory difficulty and radiological pneumonia.

Characteristic	Deaths (n = 35)	Survivors (n = 372)	RR	95% CI	p
Male sex	23 (66)	207 (56)	1.48	0.75–2.88	0.331
Age in months (median, IQR)	8.0 (4.9, 11.0)	10.0 (5.0, 18.0)	-	-	0.116
Live outside Dhaka district	11 (31)	80 (22)	1.59	0.81–3.12	0.256
Live in slum area	17 (49)	169 (45)	1.12	0.60–2.11	0.858
Poor socio-economic condition	30 (86)	314 (84)	1.10	0.44–2.72	0.968
Employed mother	10 (29)	51 (14)	2.27	1.15–4.48	0.035
Lack of intake of BCG vaccination	2 (6)	49 (13)	0.43	0.11–1.75	0.287
Lack of intake of DPT/oral polio/HIV/Hepatitis vaccination	5 (14)	77 (21)	0.66	0.26–1.65	0.494
Non-breastfed from neonatal period	5 (14)	58 (16)	0.91	0.37–2.26	0.968
Weight for length Z score	-3.7 ± 1.8	-3.8 ± 1.4	-0.09*	-0.42–0.60	0.727
Weight for age Z score	-5.6 ± 1.7	-4.9 ± 1.5	-0.64*	-1.17 –-0.12	0.016
Mid upper arm circumference	10.5 ± 1.3	10.5 ± 1.3	-0.39*	-0.89–0.13	0.141
AWD	31 (89)	275 (74)	2.56	0.93–7.07	0.087
Fever	22 (63)	210 (57)	1.28	0.66–2.46	0.580
Dehydration	16 (46)	36 (10)	5.75	3.16–10.45	<0.001
Duration of fever	3.0 (3.0, 5.0)	4.5 (3.0, 7.0)	-	-	0.103
Presence of danger signs	23 (66)	88 (24)	5.11	2.63–9.92	<0.001
Severe sepsis	6 (14)	14 (4)	4.27	1.99–9.14	0.001
Severe anaemia	0	4 (1)	-	-	1.0
Overall bacteraemia	5 (14)	13 (4)	3.58	1.58–8.14	0.014
Hypokalaemia	17 (49)	122 (33)	1.82	0.97–3.42	0.090
Hyperkalaemia	6 (17)	48 (13)	1.35	0.59–3.11	0.655
Hyponatraemia	6 (17)	56 (15)	1.15	0.50–2.66	0.934
Hypocalcaemia	17 (49)	83 (22)	2.90	1.55–5.41	0.001
Hypomagnesaemia	2 (6)	10 (3)	1.99	0.54–7.37	0.275

Figures represent n (%), unless specified. RR: relative risks. CI: confidence interval. IQR: inter-quartile range

*: mean difference

**Table 6:** Results of log-linear binomial regression, to identify independent risk factors for deaths in SAM children presenting with cough or respiratory difficulty and radiological pneumonia and who received standard WHO interventions.

Characteristic	RR	95% CI	p value
Employed mother	2.79	1.73–4.49	<0.001
Dehydration	3.16	1.81–5.51	0.001
Presence of danger signs	3.16	1.65–6.06	0.001
Severe sepsis	1.59	0.79–3.19	0.190
Hypocalcaemia	1.99	1.23–3.22	0.005
Bacteraemia	2.72	1.68–4.42	<0.001

The sensitivity, specificity, positive predicting value (PPV), and negative predictive value (NPV) of the identified risk factors for treatment failure and deaths in our study children are very poor (Table 7). However, the performance of danger signs of severe pneumonia and fever in predicting treatment failure and deaths are better compared to others (Table 7).

**Table 7:** Validity of predicting features of treatment failure and deaths in SAM children presenting with cough or respiratory difficulty and radiological pneumonia and who received standard WHO interventions.

Characteristics	Treatment failure				Deaths			
	Sensitivity (95% CI)	Specificity (95% CI)	PPV (95% CI)	NPV (95% CI)	Sensitivity (95% CI)	Specificity (95% CI)	PPV (95% CI)	NPV (95% CI)
Employed mother	29 (15–47)	86 (82–90)	16 (9–29)	93 (89–95)	15 (9–22)	85 (80–89)	30 (19–43)	93 (89–95)
Documented fever	63 (45–78)	44 (38–49)	9 (6–14)	93 (87–96)	72 (63–79)	49 (43–55)	38 (32–45)	80 (73–86)
Dehydration	46 (29–63)	90 (87–93)	31 (19–45)	95 (92–97)	22 (15–30)	91 (87–94)	52 (38–66)	73 (68–77)
Presence of danger signs	66 (48–80)	76 (72–81)	21 (14–30)	96 (93–98)	52 (43–61)	83 (78–87)	58 (48–67)	80 (75–84)
Severe sepsis	18 (8–36)	95 (92–97)	30 (13–54)	90 (86–94)	9 (5–16)	96 (94–98)	55 (32–76)	71 (67–76)
Hypocalcaemia	49 (32–66)	78 (73–82)	17 (10–26)	94 (91–96)	37 (29–46)	81 (76–85)	46 (36–56)	75 (70–80)
Bacteraemia	14 (5–31)	73 (68–76)	4 (1–9)	92 (89–95)	8 (3–15)	97 (94–99)	56 (31–78)	71 (66–75)

PPV: positive predictive value. NPV: negative predictive value.

## Discussion

In this study, we observed significantly higher treatment failure and deaths among SAM children with danger signs of severe pneumonia compared to those without the danger signs when the initial antibiotic therapy was guided by the WHO recommendation [18]. A recently published report on bacterial aetiology of pneumonia among SAM children from this same study population noted isolation of a wide range of bacterial pathogens with predominance of Gram-negative bacteria [23]. Severely malnourished children are known to be immune-compromised and thus they might fail to exhibit overt clinical signs of pneumonia due to depressed cell mediated and humoral immune responses [24] and hypokalaemia [25], and consequently danger signs of severe pneumonia in such children may become apparent only at an advanced stage of their illness [24, 26]. SAM children with such severe illness may also have higher circulating level of aflatoxin, small bowel overgrowth; and they may likely to develop gram-negative sepsis [27] that we observed in this study. Moreover, the gram negative bacteria often produce endotoxin [27]. The combination of severe infection, higher circulating aflatoxin, bacterial endotoxin, and small bowel overgrowth in SAM children may be associated with oxidative stress and endogenous production of nitric oxide [27], potentially contributing to treatment failure and death among our study children despite treatment with ampicillin and gentamicin. On the other hand, a very small number of the bacteria isolated from our study population was resistant to ampicillin and gentamicin, but were more susceptible to fluoroquinolone such as ciprofloxacin and to extended spectrum cephalosporin such as ceftriaxone. Although, our initial antibiotic therapy was guided by WHO guidelines, and were continued until further deterioration of clinical signs or when no improvement occurred even after 48 hours of therapy [18], given the apparently very small number of children with bacteria non-susceptible to ampicillin and gentamicin, and susceptible to other antibiotics, we could not claim that this delay in switching to second line antibiotic (ceftriaxone) potentially led to death.

As, only 4.4% (18/407) of our study children had bacterial isolates, it is not unexpected that a number of other important factors were potentially associated to have worse outcomes. Our further observation of association of danger signs of severe pneumonia, dehydration, hypocalcaemia and bacteraemia with treatment failure and deaths supports this speculation. A number of recent evidences revealed that poor outcomes were often found to be associated with danger signs of severe pneumonia [19], hypocalcaemia [28], dehydration [29], and bacteraemia [23]. Moreover, the observation of significant association of fever and severe sepsis with treatment failure is consistent with recent data [21]. However, we do not have any ready explanation about the observed association of employed mother with deaths in our study children. As almost all of the working mothers were slum dwellers, we speculate that these abandon children at home might had lack in care and simultaneously they might brought their ill children late in the hospital and these may lead in worsening the ongoing illness and potentially contribute to deaths. While, previous data suggest it is possible that initiation of therapy with ceftriaxone for children with danger signs of severe pneumonia at the time of hospitalisation could potentially reduce treatment failures and deaths [9, 10], from our available data it is difficult to make an inference that the poor outcomes in children with WHO-defined danger signs of severe pneumonia are due to antibiotic failure. Although, the sensitivity, specificity, PPV and NPV for most of the predictive features of treatment failure and deaths were very poor, the performance of danger signs of severe pneumonia in predicting both the poor outcomes in SAM children is reasonably better and may indicate that delay in treating the children with danger signs of severe pneumonia potentially further expedite poor outcome. Thus, a carefully conducted, randomized, controlled clinical trial evaluating the efficacy of extended spectrum cephalosporin and/or fluoroquinolone versus WHO recommended initial therapy is imperative to consolidate or refute our speculation.

Our observation of 4.4% bacterial isolates from blood culture as a cause of pneumonia in our study patients is relatively low, but is consistent with the low blood culture yield in previously published etiological data in pneumonia children [30]. More than sixteen percent children (67/407) in our study reported to have antibiotics prior to admission which might have an impact in low yield. Moreover, our observation of only 2 (2/67)% bacterial isolates from blood culture among the study children who had taken antibiotics prior to admission indicates that if blood culture in those children could be done before the use of antibiotics, the yield might be much higher. A caregiver history of antibiotic administration usually understates the true proportion of children who have been administered antibiotics prior to presentation and that might have an impact in low yield from blood. However, often intake of low volume blood (<2 ml) for culture from SAM children and lack of performing the second blood culture might also have an impact on our observation of low prevalence of bacteraemia in our study population.

The observation of significant association of fever, dehydration, severe sepsis, hypokalaemia, hypocalcaemia, and hypomagnesaemia with danger signs of severe pneumonia compared to those without danger signs in SAM children is very important to notice as all of them might act as biological basis in developing danger signs of severe pneumonia in SAM children. Dehydration in SAM children with diarrhoea and pneumonia has been associated with mixed metabolic and respiratory acidosis [29], potentially contributed in developing danger signs of severe pneumonia. Fever may be absent in pneumonic SAM children due to their poor immune-response [24]; however, oxidative stress and endogenous production of nitric oxide resulting from severe infections, including severe pneumonia, are often associated with fatal clinical syndrome such as severe sepsis that includes high fever [27]. Hypokalaemia, hypocalcaemia, and hypomagnesaemia are common in SAM children and often associated with poor mental status [24] including convulsions which is the danger signs of severe pneumonia in SAM children. This was a non-randomized, observational study in which the intervention was the same for both the groups, which is its most important limitation that precludes generalisation of our findings.

In conclusion, this study observed higher treatment failures and deaths among hospitalised, severely malnourished children under five with danger signs of severe pneumonia who received WHO recommended interventions compared to those without the danger signs. Independent risk factors for poor outcomes found to be insensitive in our study population; however, danger signs of severe pneumonia seem to be best predictor of treatment failure and deaths in SAM children presenting with cough or respiratory difficulty and radiological pneumonia. We believe that the study findings provide a strong backdrop to carefully conduct a randomized controlled clinical trial for evaluating efficacy of therapy with an extended spectrum cephalosporin and/or fluoroquinolone compared to standard WHO therapy in modulating treatment failure and deaths in such children.
